# Quantification of carotid artery plaque and peri-vascular adipose tissue attenuation on computed tomography

**DOI:** 10.1093/ehjimp/qyaf040

**Published:** 2025-04-08

**Authors:** Beth Whittington, Viswan Thiagarajah, Evangelos Tzolos, Jakub Kaczynski, Caelan Taggart, Alex Vesey, Damini Dey, Rachael O Forsythe, Andrew Tambyraja, Edwin J R van Beek, Marc R Dweck, David E Newby, Michelle C Williams

**Affiliations:** BHF Centre for Cardiovascular Science, University of Edinburgh, 47 Little France Crescent, Edinburgh EH16 4TJ, UK; BHF Centre for Cardiovascular Science, University of Edinburgh, 47 Little France Crescent, Edinburgh EH16 4TJ, UK; BHF Centre for Cardiovascular Science, University of Edinburgh, 47 Little France Crescent, Edinburgh EH16 4TJ, UK; BHF Centre for Cardiovascular Science, University of Edinburgh, 47 Little France Crescent, Edinburgh EH16 4TJ, UK; BHF Centre for Cardiovascular Science, University of Edinburgh, 47 Little France Crescent, Edinburgh EH16 4TJ, UK; BHF Centre for Cardiovascular Science, University of Edinburgh, 47 Little France Crescent, Edinburgh EH16 4TJ, UK; Department of Medicine (Division of Artificial Intelligence in Medicine) and Biomedical Imaging Research Institute, Cedars-Sinai Medical Centre, Los Angeles, CA 90048, USA; BHF Centre for Cardiovascular Science, University of Edinburgh, 47 Little France Crescent, Edinburgh EH16 4TJ, UK; The Edinburgh Vascular Service, Royal Infirmary of Edinburgh, NHS Lothian, Edinburgh EH16 4SA, UK; BHF Centre for Cardiovascular Science, University of Edinburgh, 47 Little France Crescent, Edinburgh EH16 4TJ, UK; The Edinburgh Vascular Service, Royal Infirmary of Edinburgh, NHS Lothian, Edinburgh EH16 4SA, UK; Edinburgh Imaging, Queen’s Medical Research Institute, Edinburgh EH16 4TJ, UK; BHF Centre for Cardiovascular Science, University of Edinburgh, 47 Little France Crescent, Edinburgh EH16 4TJ, UK; Edinburgh Imaging, Queen’s Medical Research Institute, Edinburgh EH16 4TJ, UK; BHF Centre for Cardiovascular Science, University of Edinburgh, 47 Little France Crescent, Edinburgh EH16 4TJ, UK; Edinburgh Imaging, Queen’s Medical Research Institute, Edinburgh EH16 4TJ, UK; BHF Centre for Cardiovascular Science, University of Edinburgh, 47 Little France Crescent, Edinburgh EH16 4TJ, UK; Edinburgh Imaging, Queen’s Medical Research Institute, Edinburgh EH16 4TJ, UK

**Keywords:** carotid atherosclerosis, peri-vascular adipose tissue attenuation, quantitative plaque analysis

## Abstract

**Aims:**

Quantitative assessment of carotid artery plaque on computed tomography (CT) may identify high-risk phenotypes associated with culprit lesions and subsequent ischaemic stroke or transient ischaemic attack.

**Methods and results:**

Carotid CT angiography was performed in 48 patients with acute ischaemic stroke or transient ischaemic attack within 21 days. Quantitative plaque assessment was performed in the proximal 6 cm of the internal and external carotid artery, distal 6 cm of the common carotid artery, and residual common carotid artery. Semi-automated quantification included assessment of non-calcified, calcified, low-attenuation, and total plaque, area and diameter stenosis, and peri-vascular adipose tissue attenuation. In 48 patients (mean age 71 ± 11 years, 67% male), 96 vessels were assessed with 30 (31%) identified as culprit vessels. Culprit internal carotid arteries had greater area [83 (65, 94) vs. 64 (55, 77)%] and diameter [56 (39, 74) vs. 32 (21, 48)%] stenosis and more non-calcified [563 (413, 965) vs. 428 (283 649) mm^3^, *P* = 0.04], low-attenuation [33.7 (6.9, 72.4) vs. 16.3 (3.35, 54.3) mm^3^, *P* = 0.01], and total [699 (455, 1057) vs. 492 (311, 809), *P* = 0.04] plaque. There was no difference in calcified plaque or peri-vascular adipose tissue attenuation between culprit and non-culprit internal carotid arteries. There were no differences in quantitative plaque or peri-vascular adipose tissue attenuation in the external carotid artery or common carotid artery.

**Conclusion:**

Carotid atherosclerotic plaque characteristics are the principal features associated with culprit plaques with little or no demonstrable relationship with calcified plaque or increased peri-vascular adipose tissue attenuation.

## Graphical Abstract

**Graphical Abstract:**
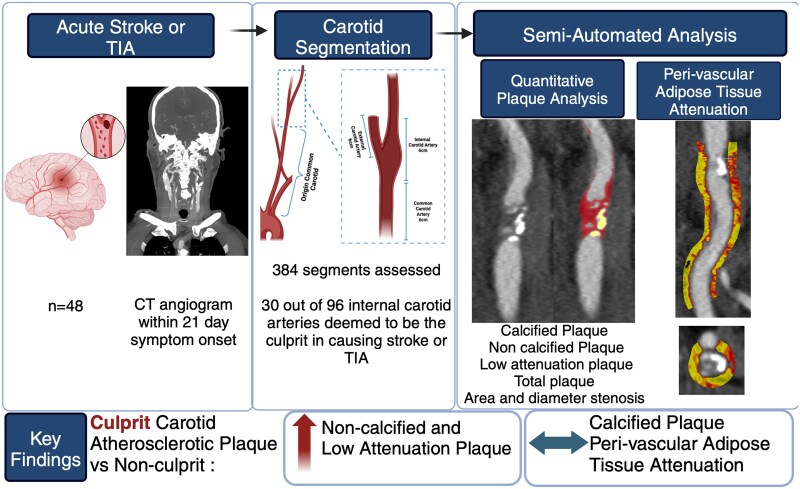

## Introduction

Assessment of the carotid arteries in patients with ischaemic stroke or transient ischaemic attack currently focuses on the degree of maximum diameter stenosis based on non-invasive imaging, predominantly assessed using ultrasound.^1^ However, more advanced assessment of the volume and composition of atherosclerotic plaque is now possible with computed tomography (CT), magnetic resonance imaging (MRI), and positron emission tomography (PET).^2,3^ In other vascular territories, such as the coronary arteries and aorta, quantitative CT assessment of atherosclerotic plaque and the peri-vascular adipose tissue attenuation can identify patients with high-risk disease subtypes which have prognostic implications.^4,5^

Based on the North American Symptomatic Carotid Endarterectomy Trial and the European Carotid Surgery Trial,^6,7^ the current routine assessment of the carotid arteries in patients with ischaemic stroke includes a 2D assessment of stenosis severity with a binary cut-off for risk stratification and consideration of carotid endarterectomy surgery.^8^ However, advances in imaging and analytical techniques mean that quantitative assessment of plaques is now possible. On CT, quantitative assessment of carotid plaque characteristics correlates with histological findings of high-risk plaque features^9^ and with cardiovascular risk scores.^10^ Calcified plaque in the carotid arteries is a common finding which is associated with adverse outcomes in some, but not all studies.^11^ Markers of carotid plaque on MRI or CT which are associated with ischaemic symptoms include a high burden of non-calcified plaque, a lipid-rich core, ulceration, and intraplaque haemorrhage.^12^ In addition, the surrounding peri-vascular adipose tissue has recently been suggested as an imaging marker of cardiovascular risk in the coronary arteries.^13^ In the carotid arteries, abnormal peri-vascular adipose tissue attenuation may be associated with symptomatic and high-risk lesions.^14–17^ To date, no studies have comprehensively assessed the association between culprit lesion identification in ischaemic stroke and quantitative measurements of CT plaque volume, characteristics, and peri-vascular adipose tissue attenuation. The aim of this study was to assess CT plaque characteristics and peri-vascular adipose tissue attenuation in culprit and non-culprit carotid plaques in patients presenting with recent acute ischaemic stroke or transient ischaemic attack.

## Materials and methods

### Study population

This was a retrospective study of 48 participants who underwent carotid CT angiography during one of three research studies. Carotid angiography was performed as part of three observational cohort studies incorporating hybrid PET and CT imaging.^3,18,19^ These studies were approved by the local Research Ethics Committee, United Kingdom Administration of Radiation Substances Advisory Committee, and local institutional review board. Written informed consent was obtained from all participants.

All participants were over 18 years old and had suffered an acute ischaemic stroke or transient ischaemic attack as defined by published guidelines and were imaged within 21 days of symptom onset.^20^ Strokes of all aetiologies were included. Exclusion criteria were impaired renal function (estimated glomerular filtration rate <30 mL/min/1.73 m^2^), inability to give informed consent or tolerate the scanner protocol, women who were pregnant or breast feeding, allergy to iodinated contrast, evidence of haemorrhagic stroke, or if participation in the study would result in a delay to carotid endarterectomy surgery.

### Clinical characteristics

Baseline clinical characteristics were obtained from study databases. The 10-year cardiovascular risk score (ASSIGN score, Assessing Cardiovascular Risk Using SIGN Guidelines to Assign Preventive Treatment) was calculated for each patient.^21^ Classification of stroke cause using the Trial of Org 10172 in acute stroke treatment (TOAST) criteria was performed by clinicians.^22^ In those deemed to have stroke secondary to large artery atherosclerosis, a culprit carotid artery was determined by the attending stroke clinician based on clinical presentation, medical records, and radiological investigations. This assessment was conducted blind to the carotid plaque and peri-vascular adipose tissue analyses performed as part of this study.

### Carotid CT angiography acquisition

Carotid CT angiography was performed on 64 or greater multi-detector CT scanners according to standard local protocols. Tube current and voltage were adjusted based on scout images, and 50–70 mL of iodine-based contrast medium was injected at 5.5–6.5 mL/s.

### Analysis of atherosclerotic plaque

Image analysis was performed blinded to clinical information. Carotid CT angiography data sets were exported in Digital Imaging and Communications in Medicine format for quantitative assessment with dedicated software (Autoplaque version 2.6, Cedars-Sinai Medical Center, Los Angeles, CA, USA).^23^ This software has been previously used for analysis of the coronary arteries with excellent observer reproducibility and validated against the gold standard of intravascular ultrasound.^24–27^

**Figure 1:**
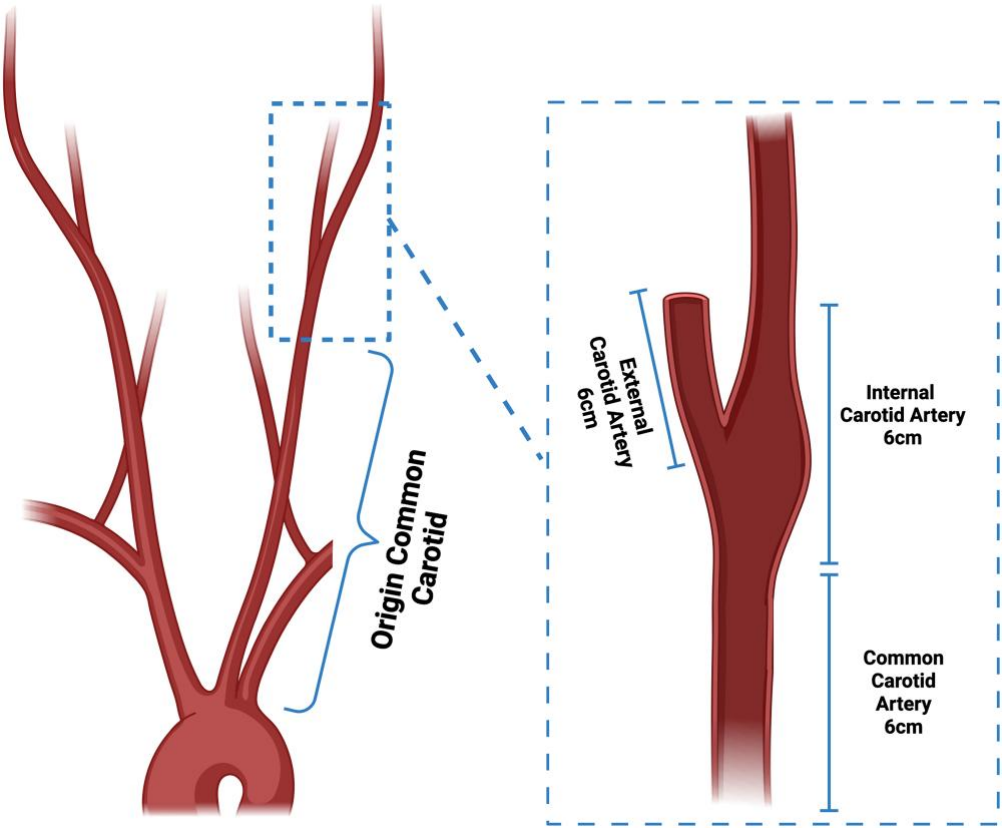
Vascular segments used for quantitative analysis. ^a^Created with BioRender.com.

Semi-automated centrelines were drawn in both carotid arteries from their origin at the arch of the aorta to the intracranial internal carotid artery. Four standardized vascular segments were assessed (*Figure 1*) defined as the proximal 6 cm of the internal carotid artery measured from the origin of the carotid bulb (the internal carotid segment), proximal 6 cm of the external carotid artery (the external carotid segment), distal 6 cm of the distal common carotid artery (the common carotid segment), and the remainder of the common carotid artery from its origin (the common carotid origin segment). A circular region of interest (10 mm diameter) was placed in the proximal aorta to serve as the normal reference blood pool attenuation.

**Figure 2:**
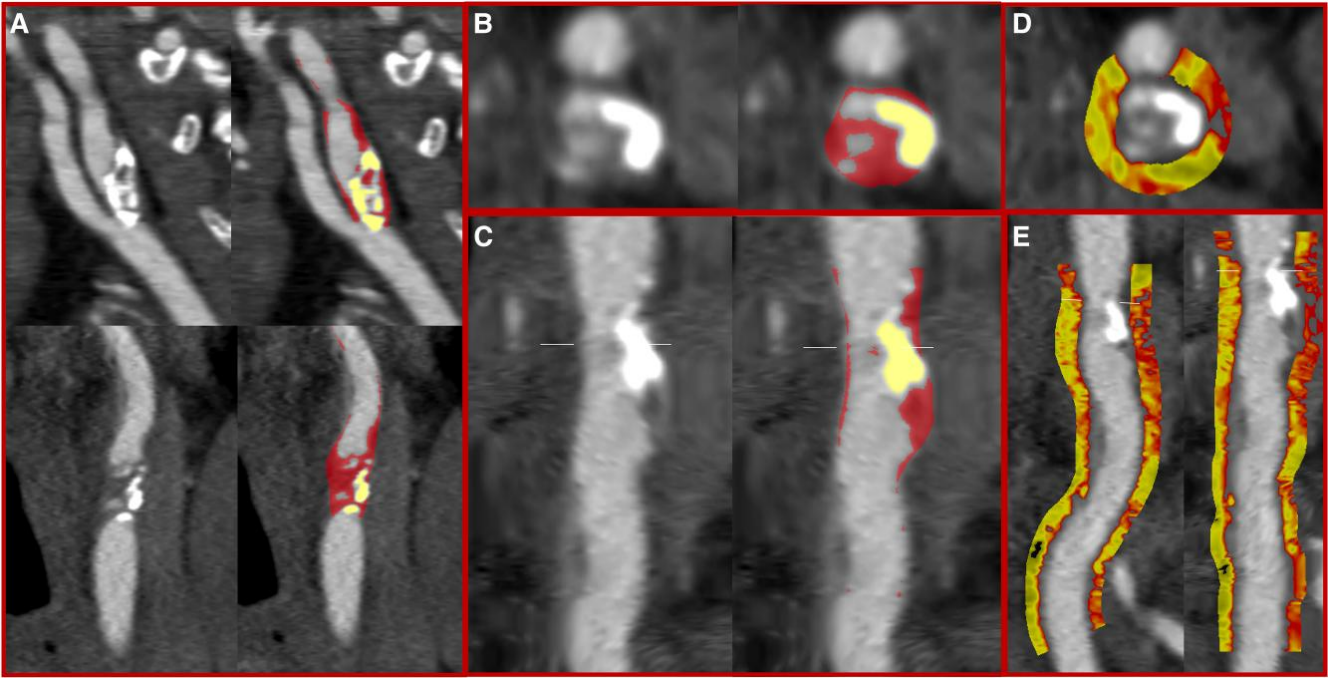
Quantitative plaque analysis. Sixty-six-year-old patient presenting with a left sided transient ischaemic attack and a 60–65% stenosis in the left internal carotid artery on Doppler ultrasound, who proceeded to carotid endarterectomy. CT showed a severe stenosis in the proximal left internal carotid artery with a high burden of calcified and non-calcified plaque on curved planar reformations (*A*), cross-sectional images (*B*), and straightened curved planar reformations (*C*). Quantitative plaque analysis shows non-calcified plaque in red (*A–C*), calcified plaque in yellow (*A–C*), and peri-vascular adipose tissue attenuation in yellow/orange (*D* and *E*).

Plaque constituents and vessel lumen were automatically contoured, with manual adjustment if required (*Figure 2*). Remodelling index (ratio of maximum vessel area to the proximal normal reference vessel area),^17^ area stenosis (maximum area stenosis compared with proximal and distal reference points, as a percentage), and maximal diameter stenosis were quantified. The volumes (in mm^3^) of total, calcified, and non-calcified plaque were measured using thresholds adjusted based on blood pool attenuation.^28^ The volume of low-attenuation non-calcified plaque was measured using a threshold of <30 Hounsfield units (HU). Plaque burden as a percentage was calculated for total and all subtypes of plaque, by dividing the plaque volume by the vessel volume of the region analysed and multiplying by 100. Per-segment analysis was performed for the common carotid, internal carotid, external carotid, and origin of common carotid artery. Segments that did not contain any atherosclerotic plaque were excluded from the per-segment analysis. A sub-analysis of only patients whose stroke was due to carotid atherosclerosis was performed which compared culprit and non-culprit internal carotid arteries in the same patient.

For combined vessel analysis, values from the internal carotid artery, distal common carotid artery, and common carotid artery origin segments were combined.

### Analysis of peri-vascular adipose tissue attenuation

Peri-vascular adipose tissue attenuation was assessed in all carotid artery segments (*Figure 1*), including those without evidence of atherosclerotic plaque. The average attenuation of all adipose tissue containing voxels with a range from −190 to −30 HU within an outer distance from the vessel wall of 3 mm was recorded. Previously defined conversion factors were used to standardize these values to account for different scanner tube voltages.^29,30^

### Statistical analysis

Statistical analysis was performed using the software package R (v4.0.2, R Foundation Statistical Computing, Vienna, Austria). Categorical variables are presented as frequencies (percentage). Data were tested for normality of distribution using the Shapiro–Wilk test. Continuous, normally distributed variable are presented as mean ± standard deviation. Non-normally distributed continuous variables are presented as median (interquartile range). Statistical significance was assessed using Pearson’s *χ*^2^ test, Wilcoxon’s signed rank test, Student’s *t-*test, one-way analysis of variance, or Mann–Whitney *U* test as appropriate. A statistically significant difference was defined as a two-sided *P*-value of <0.05.

## Results

### Study population

Forty-eight patients with a predominance of elderly men participated and were equally likely to have presented with either an acute stroke or transient ischaemic attack (*Table 1*). Thirty of the 48 patients had strokes secondary to carotid atherosclerosis. They had a high prevalence of cardiovascular risk factors and a high 10-year cardiovascular risk score with a half of patients undergoing carotid endarterectomy surgery (*Table 1*).

**Table 1:** Baseline characteristics of the study population

Baseline characteristics	Overall
Number	48
Age (years)	71 ± 11
Male	32 (67%)
Presenting syndrome	
TIA	24 (50%)
Stroke	24 (50%)
Systolic blood pressure (mmHg)	149 ± 29
Diastolic blood pressure (mmHg)	79 ± 15
Hypertension	36 (77%)
Hypercholesterolaemia	38 (81%)
Body mass index (kg/m^2^)	27 ± 17
Smoking status	
Current	12 (26%)
Ex-smoker	15 (33%)
Never smoked	19 (41%)
Atrial fibrillation	6 (13%)
Diabetes mellitus	4 (9%)
Previous stroke	11 (23%)
Previous TIA	10 (21%)
Peripheral vascular disease	6 (13%)
Coronary artery disease history	32 (69%)
Previous myocardial infarction	9 (19%)
10-year cardiovascular risk score	32 ± 18
Medications	
Anticoagulant	4 (8.5%)
DAPT	10 (21%)
Statin	40 (85%)
Subsequent endarterectomy	26 (54%)

Number (percentage %). Mean ± standard deviation.TIA, transient ischaemic attack; DAPT, dual antiplatelet therapy.

### Internal carotid artery plaque characteristics

Of the 96 internal carotid artery segments, plaque was identified in 79 (82%). Internal carotid artery segments on the culprit side had more severe diameter and area stenosis compared with the non-culprit side (*Table 2*). The culprit side also had a higher volume and burden of non-calcified, low-attenuation, and total plaque. However, there were no differences in the internal carotid artery calcified plaque or peri-vascular adipose tissue attenuation on the culprit compared with non-culprit side (*Table 2*). In the sub-analysis of only patients whose stroke was due to carotid atherosclerosis, these findings were similar (see Supplementary data online, *Table S1*).

**Table 2:** Internal carotid artery plaque characteristics in culprit and non-culprit vessels

Internal carotid plaque characteristics	Culprit (*n* = 29)	Non-culprit (*n* = 50)	*P*-value
Plaque volumes			
Non-calcified plaque volume (mm^3^)	563.46 (413.04, 965.62)	428.44 (283.45, 649.02)	**0**.**04**
Calcified plaque volume (mm^3^)	44.57 (15.02, 124.21)	36.61 (11.87, 112.39)	0.60
Low-attenuation non-calcified plaque volume (mm^3^)	33.69 (6.91, 72.40)	16.32 (3.35, 54.28)	**0**.**01**
Total plaque volume (mm^3^)	699.05 (455.14, 1057.59)	492.10 (310.59, 809.51)	**0**.**04**
Plaque burdens			
Non-calcified plaque burden (%)	36.09 (28.19, 45.89)	24.65 (18.74, 38.56)	**0**.**01**
Calcified plaque burden (%)	2.87 (0.89, 7.86)	2.87 (0.89, 7.86)	0.63
Low-attenuation non-calcified plaque burden (%)	1.95 (0.52, 4.01)	1.00 (0.17, 2.88)	**0**.**01**
Total plaque burden (%)	42 (32, 53)	27 (13.4,46.1)	**0**.**03**
Other plaque parameters			
Remodelling index	1.08 (1.01, 1.31)	1.08 (1.01, 1.26)	0.39
Maximum diameter stenosis (%)	56.31 (38.96, 74.24)	32.01 (21.14, 47.87)	**<0**.**001**
Area stenosis (%)	82.79 (64.63, 94.11)	64.50 (54.64, 77.22)	**0**.**001**
Peri-vascular adipose tissue attenuation (HU)	−63.00 (−71.83, −58.12)	−63.69 (−72.32, −58.23)	0.99

Median (interquartile range). Mean ± standard deviation. Significant differences highlighted in bold (*P*-value <0.05). HU, Hounsfield units.

### Common, external, and origin carotid artery plaque characteristics

Of the 96 segments, plaque was identified in 43 (45%) of the common carotid arteries, 26 (27%) of the external carotid arteries, and 19 (20%) of the origin of the carotid arteries. These carotid artery segments on the culprit and non-culprit sides had similar diameter and area stenoses, plaque volumes, plaque burdens, and peri-vascular adipose tissue attenuation (*Table 3*).

**Table 3:** Common carotid artery, external carotid artery, and common carotid artery origin plaque characteristics in culprit and non-culprit vessels

	Common carotid artery			External carotid artery			Common carotid artery origin		
Plaque characteristics	Culprit (*n* = 19)	Non-culprit (*n* = 24)	*P*-value	Culprit (*n* = 9)	Non-culprit (*n* = 17)	*P*-value	Culprit (*n* = 7)	Non-culprit (*n* = 12)	*P*-value
Plaque volume									
Non-calcified plaque volume (mm^3^)	300.31 (0.00, 613.28)	218.66 (66.59, 599.45)	0.94	169.13 (0.00, 311.44)	153.75 (70.86, 277.83)	0.98	621.95 (87.28, 1854.54)	272.82 (0.00, 427.58)	0.29
Calcified plaque volume (mm^3^)	9.35 (2.14, 47.65)	12.45 (3.60, 41.85)	0.90	3.66 (1.44, 9.52)	3.43 (0.75, 8.42)	0.71	41.09 (6.83, 55.82)	32.34 (3.20, 128.88)	0.78
Low-attenuation non-calcified plaque volume (mm^3^)	7.93 (0.00, 30.73)	2.89 (0.00, 22.37)	0.96	3.76 (0.00, 7.81	1.34 (0.00, 5.39)	0.66	49.05 (7.60, 216.33)	1.47 (0.00, 12.96)	0.12
Total plaque volume (mm^3^)	300.31 (62.17, 643.48)	249.94 (76.46, 659.10)	0.84	193.17 (16.79, 318.29)	163.69 (72.10, 277.83)	0.94	655.49 (129.39, 1912.47)	393.48 (43.52, 512.81)	0.349
Plaque burden									
Non-calcified plaque burden (%)	18.86 (0.00, 29.99)	9.91 (3.21, 25.16)	0.60	28.17 (0.00, 40.87)	24.64 (10.02, 36.91)	0.94	24.82 (4.54, 33.16)	11.40 (0.00, 16.35	0.252
Calcified plaque burden (%)	0.58 (0.10, 2.19)	0.50 (0.17, 2.13)	0.9	0.77 (0.25, 1.08	0.43 (0.11, 1.42)	0.61	1.07 (0.22, 1.64)	1.55 (0.17, 4.63)	0.394
Low-attenuation non-calcified plaque burden (%)	0.34 (0.00, 1.68)	0.16 (0.00, 1.22)	0.90	0.43 (0.00, 1.30	0.22 (0.00, 0.81	0.70	2.30 (0.27, 3.87)	0.06 (0.00, 0.38)	0.15
Total plaque burden (%)	20.49 (3.32, 33.52)	12.53 (3.95, 26.66)	0.57	32.17 (2.87, 41.12)	26.37 (10.19, 37.79)	0.76	25.94 (5.86, 33.80)	16.10 (4.63, 22.03)	0.30
Other parameters									
Remodelling index	1.77 (1.09, 2.48)	1.76 (1.33, 2.08)	0.93	1.10 (1.00, 1.48)	1.01 (1.00,1.18)	0.051	1.58 (1.30, 1.64)	1.02(1.01,1.12)	0.06
Area stenosis (%)	45.57 (30.48, 66.66)	39.19 (22.07, 48.58)	0.21	85.09 (63.90, 91.17)	59.08 (52.75, 71.31)	0.06	47.92 (44.56, 66.09)	42.03 (33.75, 58.23)	0.35
Maximum diameter stenosis (%)	25.73 (15.73, 40.23)	21.31 (11.70, 27.58)	0.19	58.42 (37.38, 69.69)	33.68 (29.89, 45.42)	0.06	26.00 (24.85, 40.64)	22.95 (17.87, 32.82	0.45
Peri-vascular adipose tissue attenuation (HU)	−64.9 (−72.2, −55.5)	−65.2 (−72.7, −57.9)	0.881	−65.7 (−70.2, −59.0)	−62.8 (−71.6, −54.8)	0.477	−75.6 (−82.1, −67.2)	−75.8 (−84.6, −68.3)	0.707

Median (interquartile range). Mean ± standard deviation. Analysis of peri-vascular adipose tissue attenuation includes segments without atherosclerotic plaque. HU, Hounsfield units.

### Combined vessel assessment of plaque characteristics

For combined vessel analysis, values from the internal carotid artery, distal common carotid artery, and common carotid artery origin segments were combined. Area stenosis and diameter stenosis were greater in culprit compared with non-culprit vessels.^4^ The volumes and burdens of non-calcified, low-attenuation, and total plaque were greater in culprit compared with non-culprit vessels (*Table 4*). However, there were no differences in calcified plaque or peri-vascular adipose tissue attenuation between culprit and non-culprit common vessels (*Table 4*).

**Table 4:** Per patient plaque characteristics in culprit and non-culprit vessels, including internal carotid artery, common carotid artery, and origin segments

Per patient plaque characteristics	Culprit (*n* = 30)	Non-culprit (*n* = 66)	*P*-value
Plaque volume			
Non-calcified plaque volume (mm^3^)	765 (492, 1082)	342 (0, 751)	**0**.**001**
Calcified plaque volume (mm^3^)	75 (21, 132)	24 (0, 122)	0.149
Low-attenuation non-calcified plaque volume (mm^3^)	42 (21, 87)	7.19 (0, 42.3)	**0**.**001**
Total plaque volume (mm^3^)	874 (528, 1393)	431 (0.7, 991)	**0**.**002**
Plaque burden			
Non-calcified plaque burden (%)	14 (11, 26)	7.1 (0, 17)	**0**.**001**
Calcified plaque burden (%)	1.3 (0.4, 2.7)	0.6 (0, 2.4)	0.238
Low-attenuation non-calcified plaque burden (%)	0.9 (0.4, 1.3)	0.1 (0, 089)	**0**.**001**
Total plaque burden (%)	18 (11, 30)	7.9 (0.02, 20)	**0**.**001**
Other parameters			
Remodelling index	1.42 (1.23, 1.68)	1.32 (1.18, 1.47)	0.16
Area stenosis (%)	85 (70, 96)	66 (55, 77)	**<0**.**001**
Maximum diameter stenosis (%)	56 (42 78)	37 (30, 48)	**<0**.**001**
Peri-vascular adipose tissue attenuation (HU)	−66.6 (−71.0, −61.1)	−68.1 (−75.5, −62.2)	0.672

Median (interquartile range). Mean ± standard deviation. Significant differences highlighted in bold (*P*-value <0.05). HU, Hounsfield units.

## Discussion

In this study, we used quantitative plaque analyses to assess carotid plaque characteristics and peri-vascular adipose tissue attenuation in patients presenting with ischaemic stroke or transient ischaemic attack. We showed that culprit vessels, and in particular the internal carotid artery segments, had more non-calcified, low-attenuation, and total plaque compared with non-culprit vessels. However, there were no differences in calcified plaque or the peri-vascular adipose tissue attenuation in culprit compared with non-culprit vessels. This suggests that non-calcified and low-attenuation plaques are the principal features associated with culprit carotid plaques with little or no demonstrable contribution from plaque calcification or increased peri-vascular adipose tissue attenuation.

Carotid artery imaging has in the past focused on the presence and severity of stenoses. More recently, visual assessment of the components and characteristics of atherosclerotic plaque on non-invasive imaging has been developed, with standardized imaging classifications now available.^2,12^ These qualitative assessments of carotid plaque characteristics have identified high-risk plaque features which are associated with the culprit vessel^3^ and subsequent outcomes, including recurrent ischaemic stroke and all-cause mortality.^31,32^ These high-risk plaque features include the presence of a thin fibrous cap, intraplaque haemorrhage, increased wall thickness, neovascularization, ulceration, and arterial remodelling. However, these qualitative visual assessments of carotid plaque can be time-consuming and prone to observer variability. Quantitative analysis of carotid plaque has the potential to improve standardization of these assessments on carotid CT angiography.

Quantitative plaque analysis is a relatively new addition to the assessment of carotid CT angiography. It correlates well with histological findings in carotid endarterectomy specimens^9^ including calcified, fibrotic, and fatty components of the plaque^33^ as well as cardiovascular risk scores.^10^ Our study showed that non-calcified, low-attenuation, and total plaque volumes and burdens were associated with the culprit vessel. However, calcified plaque was not associated with the culprit vessel. Non-calcified low-attenuation plaque is more unstable and prone to rupture than other plaque types, leading to clinical events of myocardial infarction and stroke from atherothrombosis of the coronary and carotid arteries respectively.^5,11^ In contrast, calcified plaque in the carotid arteries is a common finding in asymptomatic patients which increases in prevalence with age and may represent more stable disease.^25^ Indeed, meta-analyses have shown that the presence of calcified plaque was associated with the absence of ipsilateral ischaemia.^11,26^ These associations are consistent with our findings of increased non-calcified and low-attenuation plaque volume and burden in culprit vessels when compared with non-culprit vessels, with no associated increase in calcified plaque.

As well as the presence of non-calcified plaque, the site of the atherosclerotic plaque is important to consider when determining whether a plaque is a culprit. The commonest site for atherosclerotic plaque is internal carotid arteries, which gives rise to anterior circulatory ischaemic strokes and transient ischaemic attacks. However, other segments of the carotid vasculature may also contain atherosclerotic plaque and our study is the first to provide a volumetric plaque analysis for all segments of the carotid vasculature. As expected, our results showed the majority of plaque was found in the culprit internal carotid artery, whereas an increase in the high-risk low-attenuation plaque burden and volume was not demonstrated in the external, origin of the common carotid, or common carotid artery.

Recent studies have suggested that the peri-vascular adipose tissue attenuation may be a useful marker of high-risk vascular disease. In the coronary arteries, an increase in the peri-vascular adipose tissue attenuation is associated with a small increase in the risk of subsequent cardiac events, independent of other quantitative plaque characteristics.^13^ The aetiology of this association is uncertain, and although it may be related to local or systemic inflammation, associations with inflammatory markers have been inconsistent. Alternative explanations for this association include the presence of plaque ulceration or irregularities, local increases in iodinated contrast such as with microvascular dysfunction, and technical factors. Interestingly, the coronary arteries are the only vessel where this association has been convincingly been demonstrated and the strongest associations are seen with all-cause or cardiac mortality rather than myocardial infarction itself. However, in other regions of the cardiovascular system, there is not a consistent association with cardiovascular events. For example, peri-vascular adipose tissue attenuation surrounding the aortic root is not associated with aortic stenosis or its severity,^4^ and in the abdominal aorta, it is not associated with aneurysm size in asymptomatic patients with aortic aneurysm disease.^27^

A small number of previous studies have assessed the peri-vascular adipose tissue attenuation in the carotid arteries. Baradaran *et al.*^16^ found a higher peri-vascular adipose tissue attenuation around the internal carotid artery ipsilateral to the stroke or transient ischaemic attack symptoms compared with asymptomatic patients, with higher values in stenotic compared with non-stenotic segments. Zhang *et al.*^14^ similarly found that an increase in peri-vascular adipose tissue attenuation was associated with culprit carotid plaques in patients presenting with stroke or transient ischaemic attack. Two other studies demonstrated an increase peri-vascular adipose tissue attenuation with carotid intraplaque haemorrhage^15,17^ and more complex carotid artery plaque morphology, including plaques with a thin fibrous cap and lipid-rich necrotic core.^17^

In contrast, we identified no association between peri-vascular adipose tissue attenuation and the culprit vessel. There are several potential reasons for the different results in our study. We have used a dedicated quantitative analytical approach that provides volumetric analysis of the entire vessel, whereas several previous studies only used manual delineation of regions of interest from a selective single axial slice at the level of maximal internal carotid artery stenosis^16^ or high-risk plaque features.^17^ This manual method is prone to observer variability and only provides peri-vascular adipose tissue attenuation assessment at a single level which is likely to incorporate subjective biases. Two previous studies used semi-automated volumetric software, but they only assessed one carotid artery segment containing plaque or performed assessment at the site of visually assessed high-risk plaque features. In contrast, we have systematically assessed all the carotid artery segments and performed combined per vessel analysis. This is important as we know that carotid plaque may occur at several distinct sites within the carotid vasculature and that complex plaque can span several regions, particularly between the common and internal carotid arteries. The previous studies performing volumetric analysis also included a larger diameter surrounding the carotid arteries by using the radial distance from the outer vessel wall equal to the diameter of the carotid vessel which increases the chance of artefacts from non-adipose structures impacting their analysis. A strength of our study is the use of carotid artery segments contralateral to the side of stroke or transient ischaemic attack as ‘non-culprit’ which provides a robust contemporaneous and within-subject control in our study population. This provides validity to our results, along with the fact that there were no differences in peri-vascular adipose attenuation of the external carotid artery segments between the ipsilateral (culprit) and contralateral vessels (non-culprit). We therefore believe that we have provided a more comprehensive, internally cross-validated and accurate assessment of carotid artery peri-vascular adipose tissue attenuation than previous studies. It is possible that differences in the patient cohorts could account for these results, and larger studies will provide valuable additional information.

An interesting finding in our study was the regional differences in the peri-vascular adipose tissue attenuation within the different segments of the carotid artery. Regional variation has previously been observed within the coronary arteries where the left anterior descending artery has the lowest value, and these differences are not fully understood. In our study, the origin of the carotid artery had the lowest mean peri-vascular adipose tissue attenuation in our study compared with the other carotid artery segments. In a previous study, we showed that aortic peri-vascular adipose tissue attenuation in aneurysmal and non-aneurysmal segments of abdominal aorta in asymptomatic patients was lower compared with coronary artery values.^27^ This suggests that the vascular system is surrounded by different variants brown/white fat at different positions and this may have clinical implications. However, further study of peri-vascular adipose tissue attenuation across the body is required before this can be confirmed.

Our study has several limitations that should be acknowledged. First, this was a modest sized retrospective study involving three cohorts of patients who underwent imaging for research or clinical indications. Due to the cohort size and high frequency of carotid endarterectomy, the impact on outcomes cannot be assessed. We acknowledge that defining culprit plaque based on clinical judgement and degree of carotid stenosis on imaging is also a limitation. Differences in scanner and acquisition protocols can impact quantitative plaque and adipose tissue analysis. Finally, the semi-automated plaque analysis software was designed for use in the coronary arteries, and its application in the carotid arteries is emerging.

## Conclusion

We found that quantitative carotid plaque characteristics on CT angiography can be used to identify the culprit vessel in patients presenting with ischaemic stroke or transient ischaemic attack. In particular, culprit vessels had more non-calcified, low-attenuation, and total plaque volume and burden. However, calcified plaque and peri-vascular adipose tissue attenuation was not associated with the culprit vessel, suggesting that these features may have a limited role in the identification of culprit carotid plaques.

## Supplementary Material

qyaf040_Supplementary_Data

## Data Availability

The data that support the findings of this study are available from the corresponding author upon reasonable request.

## Lead author biography

Beth Whittington is a medical doctor currently engaged in her cardiology speciality training in the Royal Infirmary of Edinburgh, UK. She is currently pursuing her PhD at the University of Edinburgh since starting in 2021 funded by the British Heart Foundation. She completed her Medical Degree at the University of Aberdeen in 2014 and also obtained a Bachelor of Science Degree in Biochemistry at Queen’s University of Belfast in 2012. Her research focuses on imaging modalities in atheroembolic cardiovascular disease including computed tomography, magnetic resonance imaging, and positron emission tomography.
