# Supplementary material for: Quantification of carotid artery plaque and peri-vascular adipose tissue attenuation on computed tomography
Source: Eur Heart J Imaging Methods Pract. 2025 Apr 8;3(1):qyaf040. doi: 10.1093/ehjimp/qyaf040 (PMC12023745; doi:10.1093/ehjimp/qyaf040)
Supplement: qyaf040_Supplementary_Data [file qyaf040_supplementary_data.docx]

**Supplementary Materials**

*Supplementary Methods*

For patients with the iThrombus and MICA Studies a Biograph mCT Siemens Medical Systems CT Scanner was used. Helical computed tomography was performed with tube voltage with tube current selected automatically based on scout images. Patient that had the carotid CT angiogram preformed as part of their standard clinical care according to local CT protocols.

*Supplementary Results*

Supplementary Table 1. Internal carotid artery plaque characteristics in culprit and non-culprit vessels in patients with stroke due to carotid atherosclerosis.

| **Internal carotid plaque characteristics** | **Culprit**  **n=30** | **Non-culprit**  **n=30** | ***p* value** |
| --- | --- | --- | --- |
| **Plaque volumes** |  |  |  |
| Non-calcified plaque volume (mm^3^) | 563 [392, 940] | 392 [110,603] | **0.004** |
| Calcified plaque volume (mm^3^) | 44 [11, 117] | 29 [1.3, 92] | 0.33 |
| Low-attenuation non-calcified plaque volume (mm^3^) | 31.9 [6.6, 72.1] | 8.6 [1.3, 53.5] | **0.01** |
| Total plaque volume (mm^3^) | 666 [410, 1054] | 445 [183, 756.5] | **0.02** |
| **Plaque burdens** |  |  |  |
| Non-calcified plaque burden (%) | 35.4 [27.8,45.6] | 23.4 [6.1, 41.4] | **0.04** |
| Calcified Plaque Burden (%) | 2.8 [0.9, 7.3] | 1.9 [0.1, 5.4] | 0.45 |
| Low-attenuation non-calcified Plaque Burden (%) | 1.7 [0.5, 4.0] | 0.53 [0.1, 3.4] | **0.01** |
| Total plaque Burden (%) | 42 [32, 53] | 27 [13.4,46.1] | **0.03** |
| **Other plaque parameters** |  |  |  |
| Remodelling Index | 1.08 [1.01, 1.30] | 1.10 [1.03, 1.3] | 0.39 |
| Maximum diameter stenosis (%) | 56 (39.7,73.8) | 38 (28.6,49.1) | **0.004** |
| Area Stenosis (%) | 81.9 (65.5,93.7) | 65 (50.7, 77.7) | **0.003** |
| Peri-vascular adipose tissue attenuation (HU) | -63.5 ± 12.1 | -63.4 ± 9.9 | 0.956 |

^a^ Median [interquartile range]. Mean ± standard deviation.

^b^ Significant differences highlighted in bold (*p* value <0.05).

^c^ HU, Hounsfield units.
